# Ionomycin enhances radiosensitivity in breast cancer through reactive oxygen species generation and mitochondrial permeability transition pore opening

**DOI:** 10.4103/mgr.MEDGASRES-D-26-00016

**Published:** 2026-06-05

**Authors:** Qi Ding, Lichen Shen, Xiao Liu, Guangyu Ju, Kaiwei Wang, Shuanghu Yuan, Junchao Qian

**Affiliations:** 1 First Clinical Medical College, Anhui University of Science and Technology, Huainan, Anhui Province, China; 2 Anhui Province Key Laboratory of Medical Physics and Technology, Institute of Health and Medical Technology, Hefei Institutes of Physical Science, Chinese Academy of Sciences, Hefei, Anhui Province, China; 3 Department of Radiation Oncology, The First Affiliated Hospital of USTC, Division of Life Sciences and Medicine, University of Science and Technology of China, Hefei, Anhui Province, China; 4 Department of Radiation Oncology, Anhui Provincial Cancer Hospital, Hefei, Anhui Province, China

**Keywords:** apoptosis, breast cancer, ionomycin, mitochondria, mitochondrial dysfunction, mitochondrial permeability transition pore, MMP, oxidative stress, radiosensitivity, ROS

## Abstract

The frequent resistance of breast cancer to radiotherapy and systemic therapies has resulted in suboptimal outcomes with traditional treatment regimens. Therefore, there is an urgent need to develop more effective methods of treating breast cancer. This study concentrated on measuring the effectiveness of a combination of anti-cancer agent, ionomycin and radiotherapy on breast cancer cells. The combination of ionomycin and radiotherapy resulted in inhibition of the proliferation of breast cancer cells *in vitro* experiments, which was indicated by cell counting kit-8 and colony formation experiments. Ionomycin and radiotherapy caused Ca^2+^ overload in mitochondrial cancer cells, opening the mitochondrial permeability transition pore, inducing reactive oxygen species generation, and increasing DNA double-strand breaks. The reduction in cell viability induced by ionomycin combined with radiotherapy was significantly reversed by N-acetyl-L-cysteine (a reactive oxygen species scavenger) and cyclosporine A (mitochondrial permeability transition pore channel inhibitor). The combination therapy extended the opening of the mitochondrial permeability transition pore in breast cancer cells, causing mitochondrial dysfunction. The prolonged opening of the mitochondrial permeability transition pore has been observed to raise the levels of cytochrome c and the levels of cleaved caspase-3 protein, which eventually resulted in an escalation in the rate of apoptosis. The treatment also decreased the migration and invasion of the breast cancer cells in this combination treatment. This research indicates that ionomycin may function as an effective radiosensitizer, therefore enhancing the efficacy of radiotherapy in the treatment of breast cancer.

## Graphical abstract

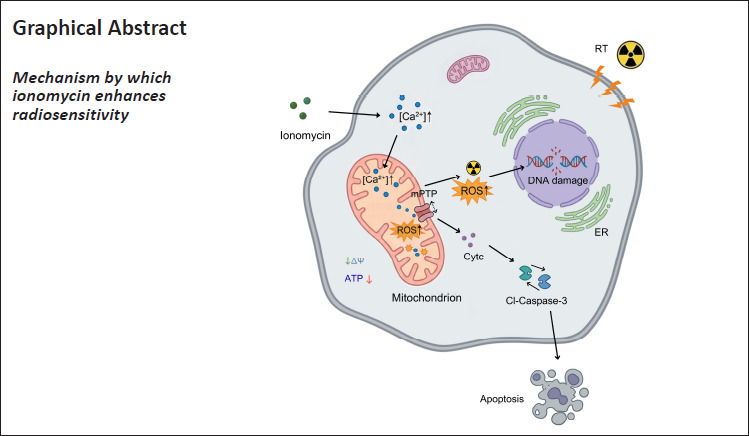

## Introduction

Breast cancer has become the most prevalent malignancy worldwide, and its incidence has been demonstrated in recent years.^1^,^2^ Among these, triple-negative breast cancer is highly invasive, rapidly progresses and easily metastasizes, accounting for 15% of all breast cancers and 25% of the mortality rate.^3^,^4^

Radiotherapy generates reactive oxygen species (ROS) through radiation-induced processes, and its therapeutic effectiveness largely depends on the efficiency of ROS production.^5^,^6^ Excessive ROS triggers downstream cellular responses, including cell cycle disruption and programmed cell death.^7^,^8^,^9^ Moreover, the activation of lymphoma-2 (Bcl-2) protein has also been observed to cause long-term reduction of ROS and radioresistance in breast cancer cells, thereby causing the limited efficiency of single radiotherapy.^10^,^11^,^12^

The mitochondrial permeability transition pore (MPTP) has been identified as a channel that crosses the mitochondrial membrane.^13^,^14^ The MPTP is opened in association with the presence of calcium in the mitochondrial matrix, especially when accompanied by oxidative stress, elevated levels of inorganic phosphates and lower concentrations of adenine nucleotides.^15^,^16^ It has been proven that opening of these channels leads to changes in the redox environments intra- and inter-mitochondrial, leading to the release of ROS.^17^,^18^ In severe oxidative stress, sustained activation of MPTP promotes the release of ROS on a grand scale, leading to irrevocable damage to the mitochondria. This fatal cascade may result in the death of cells.^19^,^20^ It has been reported that the persistent opening of the MPTP is a direct causative factor of cell apoptosis.^21^,^22^ Further, the prolonged opening of MPTP causes depolarization of mitochondrial membrane potential (MMP) and depletion of adenosine triphosphate (ATP), hence triggering mitochondrial dysfunction.^23^

Ionomycin is a calcium ion (Ca^2+^) carrier that induces the influx of extracellular Ca^2+^ and the efflux of endoplasmic reticulum Ca^2+^.^24^ Recent research has shown that ionomycin has pro-apoptotic effects on different malignancies, and this is primarily done by its capacity to impair cellular calcium metabolism by surging cytoplasmic Ca^2+^ levels.^25^ Furthermore, it has been proven that high levels of ionomycin cause an increase in the level of ROS, damage to mitochondrial function, and a decrease in ATP level.^26^

Therefore, we hypothesized that the combination of radiotherapy and ionomycin enhances cell death by inducing mitochondrial dysfunction through the MPTP. This study aimed to investigate the mechanistic basis underlying the combined treatment of cancer with radiotherapy and ionomycin and to provide a promising foundation for its therapeutic application.

## Methods

### Cell culture

Mouse breast cancer cells (4T1, Cat# STCC20022) and mouse vascular endothelial cells (C166, Cat# ORC1034) were purchased from Servicebio (Wuhan, China) and AoRuiCell (Shanghai, China), respectively, and cultured in RPMI 1640 medium (Gibco, Waltham, MA, USA) supplemented with 10% fetal bovine serum and 1% penicillin/streptomycin. Human breast cancer cells (MDA-MB-231, Cat# STCC10503) and human embryonic kidney cells (293T, Cat# STCC10301) were purchased from Servicebio and cultured in Dulbecco’s modified Eagle medium (Gibco) supplemented with 20% fetal bovine serum and 1% penicillin/streptomycin. All cell lines were incubated at 37°C in a humidified atmosphere containing 5% carbon dioxide.

### Drug/radiation treatment

N-acetylcysteine (NAC; MedChemExpress, New Jersey, NJ, USA), used as a ROS scavenger, was dissolved in phosphate-buffered saline and then diluted in RPMI 1640 medium to a final working concentration of 5 mM. Cyclosporin A (CsA; MedChemExpress), used as an MPTP inhibitor, was first dissolved in dimethyl sulfoxide and subsequently diluted in RPMI 1640 medium to a final working concentration of 20 μM.^27^ Ionomycin (Yeasen, Shanghai, China) was first dissolved in anhydrous ethanol solution. Then it was dissolved in RPMI 1640 or Dulbecco’s modified Eagle medium and diluted into different concentrations (0, 2, 4, 6, 8, and 10 μM) of ionomycin working solution and administered to cells.

**Figure 1:**
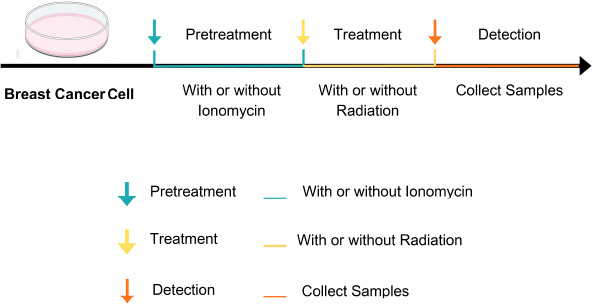
*In vitro* experimental flowchart.

For radiation treatment, the cells were irradiated (0, 4, 8, and 12 Gy) using an Elekta Infinity linear accelerator (Stockholm, Sweden). Cell cultures were placed centrally in the irradiator to receive the radiation. The experimental flowchart is shown in **Figure 1**.

### Cell proliferation and viability assay

Cell viability was assessed after treatment with ionomycin, radiotherapy, or their combination. The cells were then incubated with enhanced cell counting kit-8 reagent (Biosharp, Hefei, China) for more than 1 hour. The optical density (OD) at a wavelength of 450 nm was measured using a microplate reader (Allsheng, Hangzhou, China). Cell viability (%) = (OD_processing group_ – OD_blank group_)/(OD_negative control group_ – OD_blank group_) × 100.

For long-term proliferation analysis, cells were seeded at a density of 6 × 10³ cells/well in six-well plates (Servicebio) and cultured for 48 hours. After 2 weeks, colonies were stained with crystal violet (Servicebio), photographed, and the number of colonies was counted using ImageJ software (ImageJ 1.44p, National Institutes of Health, Bethesda, MD, USA).^28^

### Intracellular Ca^2+^ measurements

Cells were then incubated with 5 μM Fluo-3 AM (Beyotime, Shanghai, China) for 30 minutes and observed using a fluorescence microscope (Olympus, Tokyo, Japan). The mean fluorescence intensity (MFI) was quantified using the “Measure” function in ImageJ.

### Mitochondrial Ca^2+^ measurements

The cells were incubated with 5 μM Rhod-2 AM (Beyotime) for 30 minutes. A fluorescence microscope was used to carry out the observations. The ImageJ measure function was used to quantify the MFI.

### MPTP opening assay

MPTP was detected using an MPTP detection kit (Beyotime) and observed under a fluorescence microscope. When Calcein-AM enters the cell, it is cleaved by intracellular esterases, leaving it in a highly green fluorescent state within the cell. In this assay, cobalt chloride (CoCl_2_) turns off the green fluorescence by co-incubation. When CoCl_2_ cannot permeate the mitochondrial membrane and the MPTP is closed in physiological conditions, mitochondria store Calcein and are strongly green-fluorescent. On the other hand, MPTP opening allows the entry of CoCl_2_ into the mitochondrial matrix, and as a result, the green fluorescence of the mitochondrion is quenched.^29^ Hence, the degree of MPTP aperture has an inverse relationship with the magnitude of green intensity. ImageJ was used to quantify the MFI using the measure function.

### Intracellular ROS measurements

Intracellular ROS levels were assessed using 2’,7’-dichlorodihydrofluorescein diacetate (DCFH_2_-DA).^30^ The cells were incubated with 10 μM DCFH_2_-DA (MedChemExpress) for 30 minutes and observed using a fluorescence microscope. The MFI was quantified using the “Measure” function in ImageJ.

### DNA fragmentation detection

The cells were treated following the same procedure as previously described. Then the cells were fixed with 4% paraformaldehyde for 1 hour. Next, the samples were blocked with 5% bovine serum albumin at 37°C for 1 hour. Then the samples were incubated with anti-gamma H2A histone family member X (γ-H2AX) antibody (mouse, 1:1000, ZenBio, Chengdu, China, Cat# 201082-7G9, RRID: AB_2722720) at 4°C in the dark overnight, followed by incubation at room temperature for 2 hours with fluorescein isothiocyanate-conjugated goat anti-mouse IgG secondary antibody (1:1000, Enzo Life Sciences, Wuhan, China, Cat# ALX-211-200, RRID: AB_10541870). Finally, images were observed using a fluorescence microscope.

### MMP assay

MMP was determined using the JC-1 kit (Beyotime). The cells were incubated with JC-1 (2.5 μg/mL) for 30 minutes. Fluorescence images were acquired using a fluorescence microscope.

### ATP content assay

Cellular ATP content was determined by the enhanced ATP check kit (Beyotime) with the luminometer function of the Multimode microplate reader (TECAN, Männedorf, Switzerland).

### Western blotting

Cells were lysed with radio-immunoprecipitation assay buffer, and total proteins were extracted. Cellular mitochondria and cytosol were separated using a mitochondrial extraction kit (Servicebio) containing protease and phosphatase inhibitors, while cytosolic proteins were collected from the supernatant. The isolated mitochondria were then resuspended in mitochondrial lysis buffer to extract the mitochondrial proteins. Protein samples were separated by electrophoresis and transferred onto four polyvinylidene difluoride membranes which were then incubated in Tris-buffered saline with Tween buffer with 5% skimmed milk for 1 hour at 4°C and then incubated with a primary antibody against signal transducer and activator of transcription 3 (STAT3; rabbit, 1:1000, Servicebio, Cat# GB11176, RRID: AB_3094588) and phosphorylated STAT3 (rabbit, 1:1000, Servicebio, Cat# GB150001, RRID: AB_3714749), β-tubulin (rabbit, 1:1000, Servicebio, Cat# GB11017, RRID: AB_3714662), Bcl-2 (rabbit, 1:1000, Affinity, Changzhou, China, Cat# AF6139, RRID: AB_2835021), cytochrome c (Cyt c; rabbit, 1:1000, Affinity, Cat#AF0146, RRID: AB_2833328), voltage dependent anion channel 1 (VDAC-1; rabbit, 1:1000, ProteinTech, Wuhan, China, Cat# 10866-1-AP, RRID: AB_2257153) or cleaved-caspase-3 (rabbit, 1:1000, ProteinTech, Cat# 82707-13-RR, RRID: AB_3670535) at 4°C overnight. The polyvinylidene difluoride membrane was then washed three times with Tris buffered saline with Tween and incubated with a secondary antibody (goat, 1:10,000, ProteinTech, Cat# RGAR001, RRID: AB_3073505) for 1 hour at room temperature. Chemiluminescence detection (Tanon 4600, Shanghai, China) was used to obtain images. Semiquantitative analysis of protein expression was conducted via ImageJ software. Proteins of the cytoplasmic fraction use β-tubulin as the loading control, and proteins of the mitochondrial fraction use VDAC-1 as the loading control.

### Cell apoptosis assay

The cells were cultured with the Annexin V-FITC/PI apoptosis detection kit (Yeasen). Finally, apoptosis and necrosis were measured by the flow cytometer (Beckman Coulter, Pasadena, CA, USA).

### Cell scratch assay

Cells were cultured in 6-well plates at a ratio of 5 × 10^5^ cells/well. The cells were treated following the same procedure as previously described, and after 24 hours, a line scratch was made on the cell surface with the sterile pipette. The cells were then rinsed with sterile phosphate-buffered saline. The scratched cells were removed and replaced with serum-free RPMI 1640 medium. Photography was using an inverted microscope (Mshot, Guangzhou, China).

### Transwell invasion assay

The cells were treated following the same procedure as previously described. A 24-well cell culture plate was taken, and 600 μL of complete medium containing 10% fetal bovine serum was added to each well. The cell suspension was gently placed into the Transwell chamber of the PET membrane (8 μm; NEST, Wuxi, China). After 24 hours, the upper layer of the medium was discarded. Subsequently, cells were fixed with 4% paraformaldehyde for 30 minutes and stained with 0.1% crystal violet for 30 minutes. Transmembrane cells were photographed and counted under an inverted microscope. The number of invaded cells in each group was analyzed using ImageJ software.

### Statistical analysis

All statistical analyses were performed using GraphPad Prism (version 9.0; GraphPad Software, Boston, MA, USA; www.graphpad.com). All data are presented as mean ± standard deviation (SD). One-way analysis of variance was used to compare differences across groups for a single factor, followed by Dunnett post hoc test for pairwise analysis. Two-way analysis of variance was applied to evaluate the effects of two independent factors and their interaction, followed by Tukey’s *post hoc* test for pairwise analysis. A *P*-value < 0.05 was considered statistically significant.

## Results

### Concentration-dependent anti-proliferative effects of ionomycin in breast cancer cells

**Figure 2:**
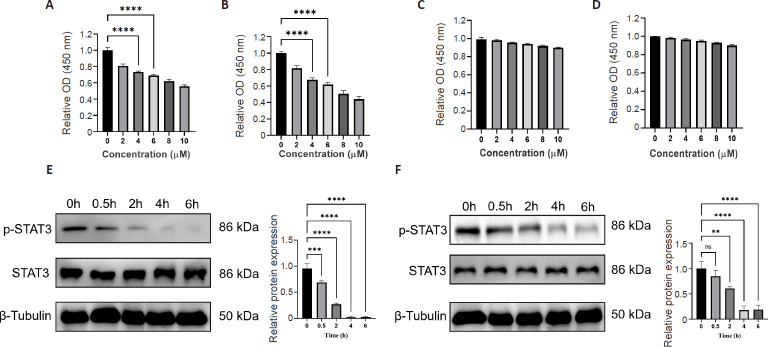
Effects of ionomycin on breast cancer cell growth. (A) Effects of ionomycin dose on 4T1 cell viability. (B) Effects of ionomycin dose on MDA-MB-231 cell viability. (C) Effects of ionomycin dose on 293T cell viability. (D) Effects of ionomycin dose on C166 cell viability. (E) Effects of ionomycin treatment at different durations on the levels of STAT3 and p-STAT3 in 4T1 cells. (F) Effects of ionomycin treatment at different durations on the levels of STAT3 and p-STAT3 in MDA-MB-231 cells. All data are presented as mean ± SD (*n* = 3). **P < 0.01, ***P < 0.001, ****P < 0.0001 (one-way analysis of variance followed by Dunnett *post hoc* test). OD: Optical density; p-STAT3: phosphorylated STAT3; STAT3: signal transducer and activator of transcription 3.

To evaluate dose-effective levels of ionomycin on the cellular proliferation *in vitro*, 4T1 and MDA-MB-231 cells were incubated under 2–10 μM ionomycin levels for 24 hours.^31^ Both cells were observed to have inhibited the growth when there was the presence of ionomycin and the growth inhibition in both cells was concentration dependent. As the concentration increased, the inhibitory effect became more pronounced. The proliferation of the two cells in the presence of ionomycin at a concentration of 4 μM was found to be inhibited. The viability of the 4T1 line decreased by 26.5% compared to the control group, while the viability of the MDA-MB-231 cell line decreased by 32.2% (**Figure 2A** and **B**). To verify the biocompatibility of ionomycin and incubate 293T and C166 cells with identical gradient concentrations of ionomycin, it was discovered that at 10 μM, 293T and C166 cells average viability remained above 80% of the control group, and within 4 μM, 293T and C166 cells average viability remained at 90% (**Figure 2C** and **D**). This result indicates that ionomycin, showing a concentration of 4 μM, has excellent biocompatibility as well as therapeutic efficacy, hence demonstrating that it can be used as a concentration for experimental purposes.

To determine the crucial time periods of growth inhibition within 24 hours, cells were treated with ionomycin for varying durations. The protein levels of STAT3 and phosphorylated STAT3 (a key regulator of cell growth^32^) were measured. The results indicated that the level of total STAT3 protein remained stable over time, whereas the level of phosphorylated STAT3 showed a slow reduction. The result of treatment of 4T1 cells with ionomycin showed a decrease in the amount of phosphorylated STAT3 after 0.5 hours of treatment and the result of MDA-MB-231 cells also showed a decrease in the amount of phosphorylated STAT3 after 2 hours. The amount of phosphorylated STAT3 was reduced after 4 hours of ionomycin treatment in both cells. The above results have shown that the least amount of treatment time needed to result in meaningful growth inhibition is 4 hours (**Figure 2E** and **F**).

### Effects of radiotherapy combined with ionomycin on breast cancer cell growth

**Figure 3:**
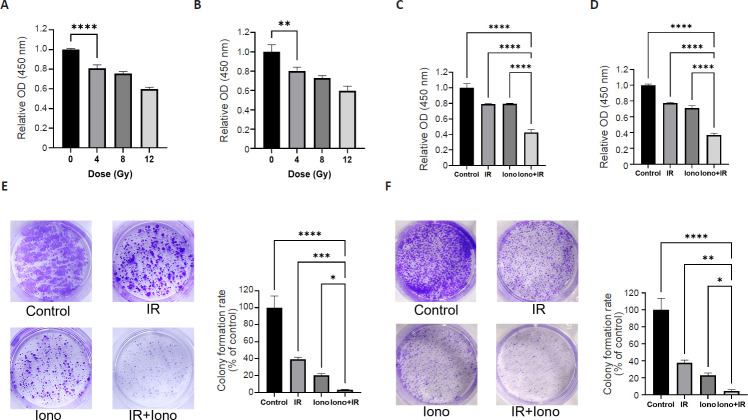
Effects of radiotherapy combined with ionomycin on breast cancer cell growth. (A) Effects of radiation does (4, 8, and 12 Gy) on 4T1 cell viability. (B) Effects of radiation does (4, 8, and 12 Gy) on MDA-MB-231 cell viability. (C) Effects of radiation (4 Gy) and ionomycin (4 μM), alone or in combination, on 4T1 cell viability. (D) Effects of radiation (4 Gy) and ionomycin (4 μM), alone or in combination, on MDA-MB-231 cell viability. (E) Effects of radiation (4 Gy) and ionomycin (4 μM), alone or in combination, on 4T1 cells colony formation. (F) Effects of radiation (4 Gy) and ionomycin (4 μM), alone or in combination, on MDA-MB-231 cells colony formation. All data are presented as mean ± SD (*n* = 3). *P < 0.05, **P < 0.01, ***P < 0.001, ****P < 0.0001 (one-way analysis of variance followed by Dunnett *post hoc* test). Iono: Ionomycin; IR: ionizing radiation; OD: optical density.

To evaluate the influence of radiotherapy on the viability of 4T1 and MDA-MB-231 cells, radiation doses of 0, 4, 8 and 12 Gy were applied, and the cells were incubated for 24 hours.^32^ Breast cancer cells subjected to the lowest dose of radiation (4 Gy) recorded a significant reduction in cell viability after 24 hours. The viability of the 4T1 cell decreased by 19.1% compared to the control group, while the viability of the MDA-MB-231 cell decreased by 19.8%. Compared with the control group, cell viability decreased in a dose-dependent manner (**Figure 3A** and **B**). In the case of both breast cancer cells, the combination of radiation (4 Gy) and ionomycin (4 μM) treatment reduced cell viability. The viability of 4T1 cells decreased to 42.5% of that of the control group, while the viability of MDA-MB-231 cells decreased to 36.9% (**Figure 3C** and **D**). The colony formation rate of both breast cancer cells was the lowest after combined treatment (**Figure 3E** and **F**). The results of this study indicate that the combination of ionomycin and radiotherapy can enhance the therapeutic efficacy of radiotherapy on breast cancer cells.

### Effects of combination therapy on MPTP opening and ROS generation

**Figure 4:**
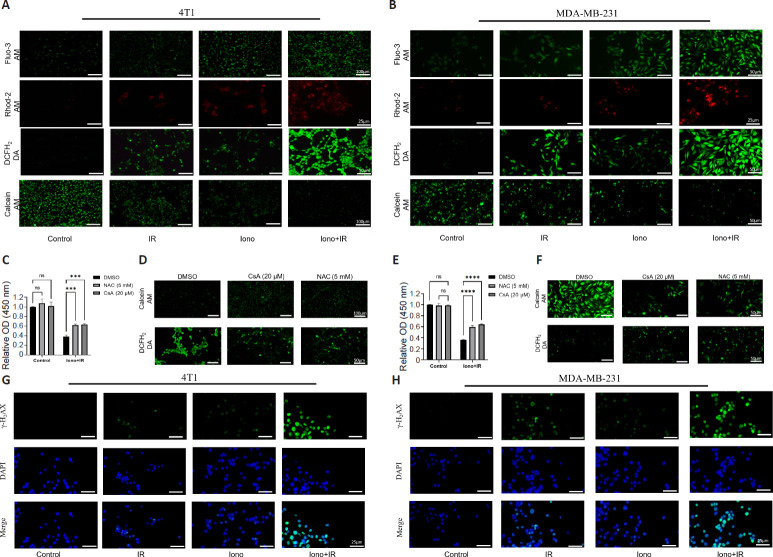
Effects of irradiation and ionomycin on MPTP opening and ROS generation in breast cancer cells. (A) Fluorescence detection using Fluo-3 AM (intracellular Ca^2+^) (Scale bar: 100 μm), Rhod-2 AM (mitochondrial Ca^2+^) (Scale bar: 25 μm), DCFH_2_-DA (intracellular ROS) (Scale bar: 50 μm), and Calcein-AM (MPTP opening) (Scale bar: 100 μm) after 24 hours treated 4T1 cells with 4 Gy irradiation, ionomycin (4 µM), or both. (B) Fluorescence detection using Fluo-3 AM (intracellular Ca^2+^), Rhod-2 AM (mitochondrial Ca^2+^), DCFH_2_-DA (intracellular ROS), and Calcein-AM (MPTP opening) after 24 hours treated MDA-MB-231 cells with 4 Gy irradiation, ionomycin (4 µM), or both. Scale bar: 50 μm. (C) CCK-8 assay of 4T1 cell viability with or without CsA (20 μM) and NAC (5 mM) pretreatment for 24 hours of co-treatment. (D) Fluorescence microscopy of changes in ROS generation (Scale bar: 50 μm) and MPTP opening (Scale bar: 100 μm) in 4T1 cells treated with or without CsA (20 μM) and NAC (5 mM) pretreatment for 24 hours of co-treatment. (E) CCK-8 assay of MDA-MB-231 cell viability with or without CsA (20 μM) and NAC (5 mM) pretreatment for 24 hours of co-treatment. (F) Fluorescence microscopy of changes in ROS generation and MPTP opening in 4T1 cells treated with or without CsA (20 μM) and NAC (5 mM) pretreatment for 24 hours of co-treatment. Scale bar: 50 μm. (G) DNA break detection of 4T1 cells with 4 Gy irradiation, ionomycin (4 µM), or both. Scale bar: 25 μm. (H) DNA break detection of MDA-MB-231 cells with 4 Gy irradiation, ionomycin (4 µM), or both. Scale bar: 25 μm. All data are presented as mean ± SD (*n* = 3). *P < 0.05, ***P < 0.001, ****P < 0.0001 (two-way analysis of variance followed by Tukey’s *post hoc* test). Calcein AM: Calcein acetoxymethyl ester; CCK-8: cell counting kit-8; CsA: cyclosporin A; DAPI: 4’,6-diamidino-2-phenylindole; DCFH_2_-DA: 2’,7’-dichlorodihydrofluorescein diacetate; Fluo-3AM: Fluo-3-pentaacetoxymethyl ester; Iono: ionomycin; IR: ionizing radiation; MPTP: mitochondrial permeable transition pore; NAC: N-acetylcysteine; OD: optical density; ROS: reactive oxygen species; γ-H2AX: gamma H2A histone family member X.

Radiotherapy induces intracellular ROS accumulation, while ionomycin, as a calcium ionophore, can elevate intracellular Ca^2+^ levels, consequently triggering MPTP opening. Both processes contribute significantly to breast cancer cell growth inhibition.^13^ In order to characterize the opening of MPTP and the generation of ROS during combination therapy, the intracellular and mitochondrial Ca^2+^ dynamics of the treatment groups were initially detected. The same changes were observed in two kinds of breast cancer cells. Compared with the control group, the intracellular Ca^2+^ concentrations were markedly elevated in both the ionomycin alone and combination groups, but increased slightly with radiotherapy alone, consistent with ionomycin’s Ca^2+^ ionophore activity. Mitochondrial Ca^2+^ staining showed moderate increases in cells treated with radiotherapy, higher increases in cells treated with ionomycin, and the highest increases in cells treated with a combination of both. These results indicate that combined treatment synergistically enhances Ca^2+^ influx into mitochondria. The analysis of the MPTP staining revealed that both radiotherapy and ionomycin mono-therapy caused the opening of pores, whereas combination therapy had the strongest effect. This is associated with the concentrations of Ca^2+^ in the mitochondria, as Ca^2+^ overload is a well-established trigger for MPTP opening.^9^ Correspondingly, ROS levels were markedly elevated in each treatment group and the most dramatic increase in the combination treatment group, consistent with MPTP-mediated ROS generation (**Figure 4A** and **B**). Analysis of the MFI also revealed that the combined treatment group induced a substantial influx of Ca^2+^, leading to a significant increase in intracellular Ca^2+^ concentration. This promoted Ca^2+^ accumulation within mitochondria and increased the opening of the MPTP, thereby enhancing ROS production (**Additional Figures 1** and **2**). In order to examine whether there is a causal relationship between ROS generation and MPTP opening, two types of breast cancer cells were exposed to either the ROS scavenger NAC (5 mM) or MPTP inhibitor CsA (20 μM), followed by combination therapy. Both interventions mitigated the decline in cell viability induced by treatment, with 4T1 cell viability increasing by 21.8% and 25%, respectively. The results of fluorescence analysis revealed that NAC pretreatment not only reduced ROS levels but also diminished MPTP opening, while CsA pretreatment suppressed both MPTP opening and subsequent ROS accumulation (**Figure 4C** and **D**). Meanwhile, MDA-MB-231 cells showed the same trend, with cell viability increasing by 23.1% and 28%, respectively. The results of fluorescence analysis also showed similar changes (**Figure 4D**, **F**, and **Additional Figures 3** and **4**). These data demonstrate that there is positive feedback between the MPTP opening and ROS generation in the combination therapy mechanism. The radiation therapy causes the DNA break, which is one of the direct pathways to kill the tumor cells and the rise in ROS levels, as an indirect method of accumulating DNA damage.^33^ We conducted immunofluorescence γ-H2AX staining in the two cells under treatment. Both samples of breast cancer cells were found to possess strong DNA break signals when subjected to combined treatments, implying that the ionomycin treatment is also involved in helping to increase the effect of radiation-induced DNA breaks in cells. This result may be related to the increase in ROS generation caused by combination therapy (**Figure 4G** and **H**).

### Effects of combination therapy on mitochondrial damage and apoptosis of breast cancer cells

**Figure 5:**
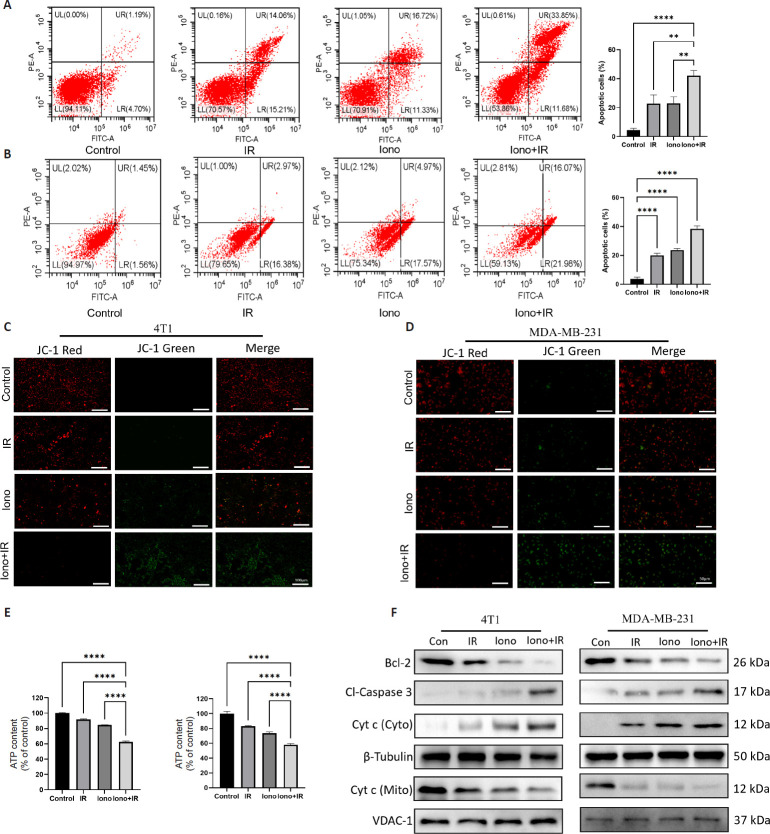
Effects of combination therapy on mitochondrial damage and apoptosis of breast cancer cell. (A, B) Apoptosis analysis of 4T1 (A) and MDA-MB-231 (B) cells after different treatments for 24 hours. (C). MMP detection in 4T1 cells following various treatments after 24 hours. Scale bar: 100 μm. (D). MMP detection in MDA-MB-231 cells following various treatments after 24 hours. Scale bar: 50 μm. (E). Determination of ATP content in 4T1 cells after different treatments for 24 hours. (F). Determination of ATP content in MDA-MB-231 cells after different treatments for 24 hours. (G). Bcl-2, Cyt c (cytoplasm and mitochondria) and cl-caspase 3 expression were tested in 4T1 and MDA-MB-231 cells following the different treatments for 24 hours. All data are presented as mean ± SD (*n* = 3). **P < 0.01, ****P < 0.0001 (one-way analysis of variance followed by Dunnett *post hoc* test). ATP: Adenosine triphosphate; Bcl-2: B cell lymphoma 2; Cl-Caspase: cleaved caspase-3; Cyt c: cytochrome C; Cyto: cytoplasm; Iono: ionomycin; IR: ionizing radiation; JC-1: 5,5’,6,6’-tetrachloro-1,1’,3,3’-tetraethyl-imidacarbocyanine iodide; Mito: mitochondrion; VDAC-1: voltage dependent anion channel 1.

Flow cytometry with Annexin V-FITC/PI staining was used as a method of analyzing apoptosis. It is important to note that the cells in the combination treatment group were highly concentrated in Q2 and Q3 compared to the other groups. For 4T1 cells, the rate of apoptosis in combination therapy is more than 40%. The combined treatment of the MDA-MB-231 cells results in an apoptosis rate of approximately 40%. This indicates that the combination treatment induced the greatest apoptotic effect (**Figure 5A** and **B**). In order to explore the MPTP-dependent process of combination therapy-induced apoptosis, we evaluated mitochondrial function through MMP and ATP production measurements. JC-1 fluorescence analysis of the two breast cancer cell lines revealed that during the time frame of radiotherapy alone, there was no loss of MMP, and with the use of ionomycin, there was relatively mild depolarization, whereas in the presence of the combination therapy, there was pronounced depolarization of MMP (**Figure 5C** and **D**). Consistent with this finding, ATP assays revealed markedly reduced cellular ATP levels following combination therapy compared to other groups, confirming mitochondrial dysfunction. Compared with the control group, the ATP content in 4T1 cells decreased by 37.7% in the combination therapy group (**Figure 5E**). The ATP content of the MDA-MB-231 cells in the combination therapy group was 57.9% of the ATP content of the control group (**Figure 5F**). The Western blot analysis performed on subcellular fractions demonstrated that 4T1 and MDA-MB-231 cells indicated the same trend. Combined treatment decreased the amount of Cyt c protein in the mitochondrial fraction with a concomitant increase in the cytosolic fraction, indicating enhanced Cyt c release from mitochondria. Moreover, an analysis of the apoptotic markers by western blotting revealed that combination treatment distinctly reduced the Bcl-2 protein expression and enhanced the caspase-3 protein cleavage (**Figure 5G**, and **Additional Figures 5** and **6**). Collectively, these findings prove that, with combination therapy, a long-lasting MPTP opening induces the mitochondrial impairment resulting in the activation of the intrinsic apoptotic pathway.

### Effects of combination therapy on the migration and invasion of breast cancer cells

**Figure 6:**
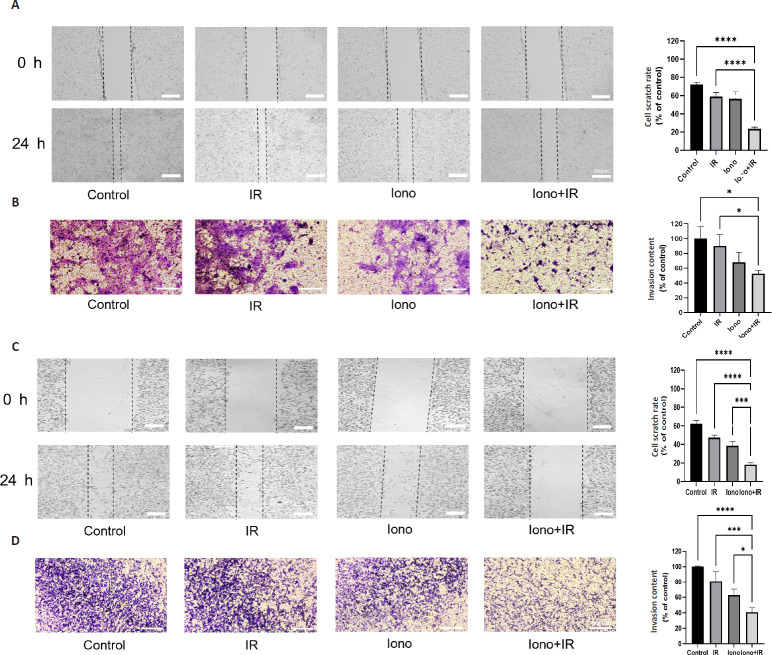
Effects of combination therapy on migration and invasion of breast cancer cells. (A) Cell scratch assay of 4T1 cells following treatment with 4 Gy irradiation, ionomycin (4 µM), or both after 24 hours. Scale bar: 200 μm. (B) Invasion assay of 4T1 cells after 24 hours. Scale bar: 50 μm. (C) Cell scratch assay of MDA-MB-231 cells. Scale bar: 200 μm. (D) Invasion assay of MDA-MB-231 cells after 24 hours. Scale bar: 100 μm. All data are presented as mean ± SD (*n* = 3). *P < 0.05, ***P < 0.001, ****P < 0.0001 (one-way analysis of variance followed by Dunnett *post hoc* test). Iono: Ionomycin; IR: ionizing radiation; ns: non-significant.

We further investigated the anti-metastasis potential of combination therapy for breast cancer by evaluating the ability of cell migration and invasion *in vitro*. In 4T1 cells, cell scratch assays showed that treatment with either radiotherapy or ionomycin alone resulted in only a modest inhibition of wound closure at 24 hours. In contrast, the combination of both treatments led to a marked suppression of cell migration (**Figure 6A**). Furthermore, invasion assays revealed that, of all the treatment groups, the combination therapy most effectively suppressed invasive capacity, reducing it by 47.5% compared to the control group (**Figure 6B**). This same trend is also observed in MDA-MB-231 cells. The cell scratch rate was 62.1% in the control group of MDA-MB-231 cells, while it decreased to 18.2% in the combination therapy group (**Figure 6C**). The invasion rate of MDA-MB-231 cells in the combination therapy group decreased to 40.7% of that in the control group (**Figure 6D**). Overall, the ionomycin combined treatment group exerted potent antimetastatic activity both horizontally and vertically.

## Discussion

In this study, we identify ionomycin as a potent radiosensitizer in breast cancer cells, acting through perturbation of calcium homeostasis and the establishment of a positive feedback loop between MPTP opening and ROS generation. This enhanced radiation sensitivity is achieved at relatively low radiation doses, which increases anti-tumor efficacy while reducing toxicity. Below, we critically examine and discuss the underlying mechanism of this synergistic effect. We also compare it with existing radiosensitization strategies and consider the translational significance and limitations of our findings. The interaction between calcium overload, MPTP opening and ROS production is central to the mechanism by which ionomycin radiosensitizes cells. Our research results suggest that ionomycin works in conjunction with radiation to encourage the sustained accumulation of calcium in the cytoplasm and mitochondria. This finding is consistent with previous studies indicating that Ca^2+^ carriers can disrupt the capacity of mitochondria to buffer calcium, thereby making cells susceptible to stress-induced death.^34^,^35^,^36^ Notably, the observation that either the ROS scavenger NAC or the MPTP inhibitor CsA abrogates the cytotoxic effects suggests a self-amplifying loop: radiation-induced ROS promote MPTP opening, which in turn facilitates further ROS release, thereby sensitizing cancer cells to subsequent radiation damage. This positive feedback mechanism differs from conventional radiosensitizers, which typically act through single-pathway modulation, and it may explain the enhanced efficacy observed at relatively low radiation doses.^37^,^38^

Beyond the immediate cytotoxic effects, our data indicate that this combined treatment engages the mitochondrial apoptotic pathway. The increased cytosolic release of cytochrome c, coupled with activation of cleaved caspase-3 and downregulation of Bcl-2, is consistent with the canonical mitochondrial pathway of apoptosis.^39^,^40^ However, prolonged MPTP opening can also trigger mitochondrial dysfunction, including loss of membrane potential and ATP depletion, which may predispose cells to necrotic or necroptotic cell death.^41^,^42^ The main modes of cell death vary depending on cell type or radiation dose, and further research methods are needed to determine the relative contribution of apoptosis to other forms of cell death.

The significant reduction in migration and invasion observed in triple negative breast cancer cells is particularly noteworthy. Triple negative breast cancer is characterized by a high metastatic potential and resistance to conventional therapies, including radiotherapy.^43^,^44^,^45^ Our findings suggest that ionomycin-mediated radiosensitization may also attenuate metastatic traits, an effect that is not commonly associated with standard radiosensitizers. While the precise mechanisms underlying this anti-metastatic effect remain to be elucidated. Future studies incorporating *in vivo* metastasis models will be essential to determine whether this translational potential holds in the complex tumor microenvironment.

Several limitations should be acknowledged in this study. First, although our data support a model in which MPTP opening and ROS generation drive the radiosensitizing effect, we cannot exclude the involvement of other calcium-dependent signaling pathways, such as calcineurin/nuclear factor of activated T cells activation or calcium-induced alterations in mitochondrial metabolism, which may contribute to the overall antitumor activity.^38^ Second, this study was conducted exclusively in breast cancer cell lines (4T1 and MDA-MB-231) under *in vitro* conditions. The tumor microenvironment, characterized by hypoxia, nutrient deprivation, and immune cell interactions, can profoundly influence both the efficacy of radiotherapy and the response to calcium-modulating agents.^39^ Therefore, validation in orthotopic animal models and patient-derived xenografts is necessary to assess the translational relevance of this combination strategy. Finally, although our preliminary assessment suggests good biosafety at the concentrations used, a comprehensive *in vivo* toxicological assessment is needed before clinical consideration.

In summary, this study demonstrates that ionomycin acts as a radiosensitizer in breast cancer cells by exploiting calcium-driven, ROS-mediated positive feedback mechanisms that converge on MPTP. The combination achieves robust antitumor effects at low radiation doses and shows promise in mitigating metastatic phenotypes in triple negative breast cancer. Future research should focus on validating these findings *in vivo*, elucidating the precise cell death pathways engaged, and exploring the applicability of this strategy to other tumor types.

## Additional files:

***Additional Figure 1:*** *Fluorescence quantification of 4T1 cells in different treatments.*

Additional Figure 1 Fluorescence quantification of 4T1 cells in different treatments.
(A) MFI of Rhod-2 AM. (B) MFI of Fluo-3 AM. (C) MFI of DCFH2-DA. (D) MFI of calcein-AM. All data are presented as mean ± SD (*n* = 3). ^*^P < 0.05, ^**^P < 0.01, ^***^P < 0.001, ^****^P < 0.0001 (one-way analysis of variance followed by Dunnett *post hoc* test). Calcein AM: Calcein acetoxymethyl ester; DCFH2-DA: 2',7'-dichlorodihydrofluorescein diacetate; Fluo-3AM: Fluo-3-pentaacetoxymethyl ester; Iono: ionomycin; IR: ionizing radiation; MFI: mean fluorescence intensity; ns: non-significant.

***Additional Figure 2:*** *Fluorescence quantification of MDA-MB-231 cells in different treatments.*

Additional Figure 2 Fluorescence quantification of MDA-MB-231 cells in different treatments.
(A) MFI of Rhod-2 AM. (B) MFI of Fluo-3 AM. (C) MFI of DCFH2-DA. (D) MFI of calcein-AM. All data are presented as mean ± SD (*n* = 3). ^*^P < 0.05, ^**^P < 0.01, ^****^P < 0.0001 (one-way analysis of variance followed by Dunnett *post hoc* test). Calcein AM: Calcein acetoxymethyl; DCFH_2_-DA: 2',7'-dichlorodihydrofluorescein diacetate; Fluo-3AM: Fluo-3- pentaacetoxymethyl ester; MFI: mean fluorescence intensity.

***Additional Figure 3:*** *Fluorescence quantification of 4T1 cells treated with different inhibitors.*

Additional Figure 3 Fluorescence quantification of 4T1 cells treated with different inhibitors.
(A) MFI of Calcein-AM. (B) MFI of DCFH2-DA. All data are presented as mean ± SD (*n* = 3). ^*^P < 0.05, ^**^P < 0.01, ^***^P < 0.001 (one-way analysis of variance followed by Dunnett *post hoc* test). Calcein AM: Calcein acetoxymethyl; CsA: cyclosporin A; DCFH2-DA: 2',7'-dichlorodihydrofluorescein diacetate; MFI: mean fluorescence intensity; NAC: Nacetylcysteine.

***Additional Figure 4:*** *Fluorescence quantification of MDA-MB-231 cells treated with different inhibitors.*

Additional Figure 4 Fluorescence quantification of MDA-MB-231 cells treated with different inhibitors.
(A) MFI of calcein-AM. (B) MFI of DCFH2-DA. All data are presented as mean ± SD (*n* = 3). ^*^P < 0.05, ^**^P < 0.01, ^****^P < 0.0001 (one-way analysis of variance followed by Dunnett *post hoc* test). Calcein AM: Calcein acetoxymethyl; CsA: cyclosporin A; DCFH_2_-DA: 2',7'-dichlorodihydrofluorescein diacetate; MFI: mean fluorescence intensity; NAC: Nacetylcysteine.

***Additional Figure 5:*** *Quantify protein expression in 4T1 cells with different treatments.*

Additional Figure 5 Quantification of protein expression in 4T1 cells with different treatments.
All data are presented as mean ± SD (*n* = 3). ^*^P < 0.05, ^***^P < 0.001, ^****^P < 0.0001 (one-way analysis of variance followed by Dunnett *post hoc* test). Bcl-2: B cell lymphoma 2; Cl-Caspase: cleaved caspase-3; Cyt c: cytochrome C; Cyto: cytoplasm; Iono: ionomycin; IR: ionizing radiation; Mito: mitochondrion; VDAC-1: voltage dependent anion channel 1.

***Additional Figure 6:*** *Quantify protein expression in MDA-MB-231 cells with different treatments.*

Additional Figure 6 Quantification of protein expression in MDA-MB-231 cells with different treatments.
All data are presented as mean ± SD (*n* = 3). ^*^P < 0.05, ^**^P < 0.01, ^****^P < 0.0001 (one-way analysis of variance followed by Dunnett post hoc test). Bcl-2: B cell lymphoma 2; Cl-Caspase: cleaved caspase-3; Cyt c: cytochrome C; Cyto: cytoplasm; Iono: ionomycin; IR: ionizing radiation; Mito: mitochondrion; VDAC-1: voltage dependent anion channel 1.

## Data Availability

*All data generated or analyzed in this study are included in this published article and its additional files.*
